# Association Between Caregiver Strain and Emergency Health Care Resource Utilization in Survivors of Critical Illness

**DOI:** 10.1016/j.chest.2024.08.057

**Published:** 2024-10-03

**Authors:** Christie Docherty, Martin Shaw, Cheuk Yu Chim, Pamela MacTavish, Helen Devine, Peter O’Brien, Phil Lucie, Lucy Hogg, Laura Strachan, Tara Quasim, Joanne McPeake

**Affiliations:** a School of Medicine, Dentistry and Nursing; University of Glasgow; Glasgow, Scotland; b Clinical Physics; NHS Greater Glasgow and Clyde; Glasgow, Scotland; c Intensive Care Unit; Glasgow Royal Infirmary; Glasgow, Scotland; d Intensive Care Unit; University Hospital Crosshouse; Kilmarnock, Scotland; e Intensive Care Unit; University Hospital Wishaw; North Lanarkshire, Scotland; f Intensive Care Unit; Victoria Hospital; Kirkcaldy, Scotland; g Intensive Care Unit; Queen Elizabeth University Hospital; Glasgow, Scotland; h The Healthcare Improvement Studies Institute, University of Cambridge; Cambridge, England

To the Editor:

After critical illness, survivors can develop new or worsening impairments, impacting their physical, mental, and cognitive health.^1^ Informal caregivers are also known to experience challenges and can experience caregiver burden and psychological distress after hospital discharge.^2^^,^^3^ There is limited evidence about the association between the challenges which informal caregivers face and patient health care utilization in the year after hospital discharge.

The aim of this study was to explore the association between caregiver strain and the number of emergency department (ED) presentations and hospital readmissions in the year after critical illness.

Ethical approval was granted by the Northwest (Liverpool Central) research ethics committee (reference No. 17/NM/0199). All participants provided consent.

This study is a secondary analysis of a prospective multicenter cohort study. All participants attended a critical care recovery program called the Intensive Care Syndrome: Promoting Independence and Return to Employment (InS:PIRE) program. Full details of InS:PIRE have been published previously.^4^^,^^5^ Briefly, InS:PIRE is an integrated health and social care intervention, delivered weekly for 5 weeks, with return visits at 3 and 12 months. Patients received a mixture of individual and group sessions with health and social care professionals (eg, welfare advice advisors, nurses, physicians, pharmacists, physiotherapists). Informal caregivers could also access support via community organizations integrated within the program. Patients and informal caregivers were recruited between 2016 and 2020 at their initial intervention appointment 4 to 12 weeks after hospital discharge. The cohort included was admitted to critical care both before and during the COVID-19 pandemic. The InS:PIRE intervention was delivered virtually during the COVID-19 pandemic.^5^

Inclusion criteria were the following: patients receiving multiple organ support and/or invasive respiratory support, patients receiving > 7 days of single organ support or postoperative care, and patients deemed by clinicians to be at high risk of postintensive care syndrome. Exclusion criteria were the following: patients who were terminally ill, patients who had suffered a traumatic brain injury, patients who remained an inpatient under psychiatric services, or patients who were incarcerated in prison.

Informal caregiver inclusion criteria were the following: the availability of paired consented patient data and caregiving responsibilities occurred in an informal, nonpaid basis. Additional exclusion criteria for this secondary analysis included patients for whom baseline data had not been obtained and patients for whom ED attendance and hospital readmission data could not be accessed.

Strain was assessed at recruitment using the Caregiver Strain Index (CSI), a 13-point instrument designed to measure the subjective burden of caregivers.^6^ A score of ≥ 7 indicates a positive screen for caregiver strain.^6^ The number of patient ED attendances and hospital readmissions within the first-year after hospital discharge were obtained via electronic medical records. Hospital admissions for planned procedures were not included in this analysis.

Analysis was undertaken using R software (R Foundation for Statistical Computing). Negative binomial regression was used to evaluate the relationship between caregiver strain and the number of ED attendances and hospital readmissions. Univariate regression was used to identify confounding variables alongside clinical domain knowledge. Both models were adjusted for age and Scottish Index of Multiple Deprivation quintile, a combined measure of social deprivation based on seven domains.^7^ Missing data were imputed using the Multivariate Imputation by Changed Equation software package.^8^

A total of 768 patients were invited to take part in the critical care recovery program, with 375 attending. In total, 299 patients were recruited to participate in research, and paired informal caregiver consent was provided for 183 patients. Fifty-nine patients were excluded because electronic ED attendances and readmission data were not available. Therefore, 124 patients and informal caregivers were included in this secondary analysis.

In the final cohort, 71 patients (57%) were male, and the median age was 59 (interquartile range [IQR], 47-66) years. In total, 18 patients (15%) included had been admitted to critical care with a diagnosis of COVID-19 pneumonitis. Most informal caregivers were female (65%). The median age of the informal caregivers was 58 (IQR, 43-64) years. Patient and informal caregiver demographics are displayed in Table 1. The median CSI score at study recruitment was 4 (IQR, 0-7).

**Table 1:** Patient and Informal Caregivers Demographics, Stratified by Caregiver Strain of Score ≥ 7 or < 7

Characteristic	Overall (N = 124)	Caregiver Strain Index		*P* Value
		< 7 (n = 90)	≥ 7 (n = 34)	
Patient demographics				
Age, y	59 (47, 66)	59 (47, 66)	57 (45, 66)	.60
Sex				.20
Male	71 (57)	48 (53)	23 (68)	
Female	53 (43)	42 (47)	11 (32)	
ICU length of stay, d	12 (7, 19)	12 (8, 20)	11 (6, 17)	.20
Hospital length of stay, d	29 (17, 51)	27 (17, 49)	32 (19, 53)	.90
APACHE II score	19 (15, 25)	19 (15, 24)	20 (12, 28)	.70
Respiratory support	114 (92)	83 (92)	31 (91)	> .90
Cardiovascular support	99 (80)	75 (83)	24 (71)	.11
Renal replacement therapy	30 (24)	21 (23)	9 (26)	.70
No. of comorbidities	2.00 (1.00, 3.00)	2.00 (1.00, 3.00)	2.00 (1.00, 4.00)	.085
Surgery at admission or within 7 d of ICU	40 (32)	31 (34)	9 (26)	.40
SIMD quintile				.50
1 (most deprived)	48 (39)	30 (33)	18 (53)	
2	27 (22)	21 (23)	6 (18)	
3	18 (15)	14 (16)	4 (12)	
4	14 (11)	11 (12)	3 (8.8)	
5 (least deprived)	17 (14)	14 (16)	3 (8.8)	
Caregiver demographics				
Age, y	58 (43, 64)	58 (43, 66)	55 (46, 61)	.40
Sex				.14
Male	40 (32)	33 (37)	7 (21)	
Female	80 (65)	55 (61)	25 (74)	
Unknown	4 (3.2)	2 (2.2)	2 (5.9)	
Relationship				.13
Partner/spouse	82 (66)	64 (71)	18 (53)	
Parent	11 (8.9)	7 (7.8)	4 (12)	
Offspring	16 (13)	10 (11)	6 (18)	
Sibling	9 (7.3)	7 (7.8)	2 (5.9)	
Friend	2 (1.6)	1 (1.1)	1 (2.9)	
Unknown	4 (3.2)	1 (1.1)	3 (8.8)	

Values are No. (%), median (quartile 1, quartile 3), or as otherwise indicated. APACHE = Acute Physiology and Chronic Health Evaluation; ICU = intensive care unit; SIMD = Scottish Index of Multiple Deprivation.

In total, there were 125 ED attendances and 81 hospital readmissions across the cohort. Three patients required a critical care admission during their hospital readmission and two patients died during the 1-year follow-up period. There was a 7.5% increase in the number of ED attendances for every 1-point increase in CSI score (95% CI, 0.2-15.4; *P* = .04), and there was a 9.9% increase in the number of emergency hospital readmissions for every 1-point increase in CSI score (95% CI, 1.0-19.5; *P* = .03) (Fig 1).

**Figure 1:**
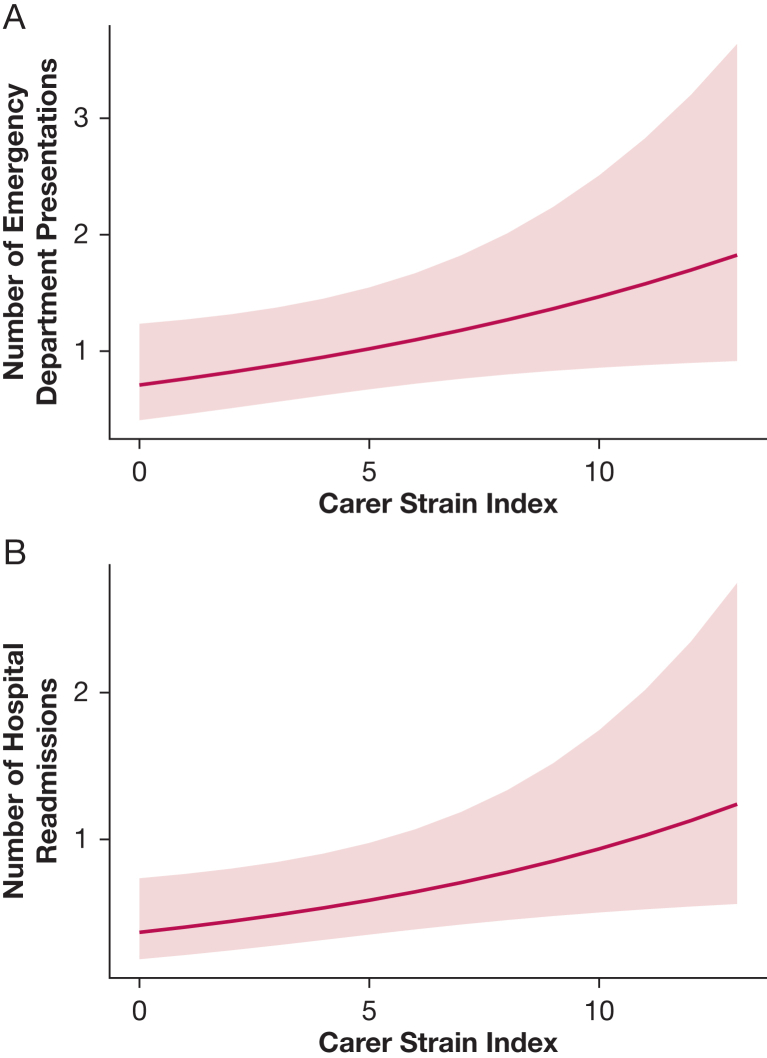
A, Number of emergency department presentations in the year after hospital discharge in relation to the Caregiver Strain Index. B, Number of hospital readmissions in the year after hospital discharge in relation to the Caregiver Strain Index. The curved line represents the absolute number of modeled attendances, and the gray band represents the 95% CI.

Across the study, 21 patients (62%) whose informal caregiver had a CSI score ≥ 7 had an ED presentation vs 39 (43%) in those patients whose informal caregiver had a CSI score < 7. The median number of ED presentations for those patients whose informal caregiver had a CSI score ≥ 7 was 1 (IQR, 0-2) vs 0 (IQR, 0-1) in those patients whose informal caregiver had a CSI score < 7.

This study suggests that caregiver strain could be associated with increased health care resource utilization in critical care survivors. This is consistent with other fields (eg, dementia), where there is an established association between caregiver status and ED use.^9^ Previous research has highlighted the high health care burden which survivors of critical illness experience after hospital discharge. To date, risk stratification of those at greatest risk of readmission has focused on areas such as acute illness trajectory and comorbidity.^10^ This analysis suggests that future research should consider the broader social context, including the needs of informal caregivers.

There are limited data on how informal caregivers can be supported, with interventions, predominantly focused on psychological distress, both in critical care and after hospital discharge, showing limited benefit.^11^^,^^12^ However, there has been limited focus on issues which address the full spectrum of health and social care problems which informal caregivers encounter.^3^ Future research should include interventions which address these crucial needs.

Key strengths of this study are that it uses data from a prospective multicenter study. There are notable limitations including the inability to access electronic health records for several patients. We do not have details of the functional status of patients at hospital discharge, which is likely to be a potential unmeasured confounder. Moreover, two patients died during follow-up; therefore, the number of hospital readmissions may have been underrepresented. Finally, only patients who attended a specific recovery program in a single hospital system were included. Other family units may have different experiences across international contexts. People who were involved in this program are also likely to have had greater resources and support, as such the use of ED resources is likely to be much higher than reported here.

In conclusion, addressing caregiver strain for informal caregivers after hospitalization could be a mechanism by which health care systems make improvements. Further research examining how optimal support for informal caregivers can be delivered is urgently needed.

## Funding/Support

This study was funded by a grant from the Health Foundation (UK).

## Financial/Nonfinancial Disclosures

The authors have reported to *CHEST* the following: J. M. was funded via a fellowship from The Healthcare Improvement Studies Institute (University of Cambridge (PD-2019-02-16). J. M.’s institution received consultancy fees from AstraZenecca for work undertaken by J. M. None declared (C. D., M. S., C. Y. C., P. M., H. D., P. O., P. L., L. H., L. S., T. Q).
